# Centromere Plasmid: A New Genetic Tool for the Study of *Plasmodium falciparum*

**DOI:** 10.1371/journal.pone.0033326

**Published:** 2012-03-30

**Authors:** Shiroh Iwanaga, Tomomi Kato, Izumi Kaneko, Masao Yuda

**Affiliations:** Mie University, School of Medicine, Tsu, Japan; Université Pierre et Marie Curie, France

## Abstract

The introduction of transgenes into *Plasmodium falciparum*, a highly virulent human malaria parasite, has been conducted either by single crossover recombination or by using episomal plasmids. However, these techniques remain insufficient because of the low transfection efficiency and the low frequency of recombination. To improve the genetic manipulation of *P. falciparum*, we developed the centromere plasmid as a new genetic tool. First, we attempted to clone all of the predicted centromeres from *P. falciparum* into *E. coli* cells but failed because of the high A/T contents of these sequences. To overcome this difficulty, we identified the common sequence features of the centromere of *Plasmodium* spp. and designed a small centromere that retained those features. The centromere plasmid constructed with the small centromere sequence, pFCEN, segregated into daughter parasites with approximately 99% efficiency, resulting in the stable maintenance of this plasmid in *P. falciparum* even in the absence of drug selection. This result demonstrated that the small centromere sequence harboured in pFCEN could function as an actual centromere in *P. falciparum*. In addition, transgenic parasites were more rapidly generated when using pFCEN than when using the control plasmid, which did not contain the centromere sequence. Furthermore, in contrast to the control plasmid, pFCEN did not form concatemers and, thus, was maintained as a single copy over multiple cell divisions. These unique properties of the pFCEN plasmid will solve the current technical limitations of the genetic manipulation of *P. falciparum*, and thus, this plasmid will become a standard genetic tool for the study of this parasite.

## Introduction

*Plasmodium falciparum* causes the most severe form of malaria in humans, and infection with this parasite is responsible for considerable morbidity and mortality worldwide. Despite intense research efforts, no effective vaccines are currently available to prevent malaria, and the global spread of drug-resistant parasites has not been halted [1].

In the post-genomic era, the genetic manipulation of *P. falciparum* is an essential technology to confront the serious problems of malaria [2], [3]. In particular, the ability to introduce transgenes is a fundamental technique in the genetic manipulation in *P. falciparum*; currently, transgenes are introduced by integration into the parasite genome by single crossover recombination [4]. This approach is widely used but still involves technical limitations because single crossover events rarely occur; it usually takes several months (e.g., 2∼6 months) to generate stable transgenic parasites. In addition, these transgenic parasites often lose the integrated transgenes during long-term cultivation as the result of additional recombination and, thus, revert to wild-type parasites. As an alternative approach, an episomal plasmid can be used for the introduction of a transgene [2], but such plasmids readily form large concatemers during replication, resulting in an increase number of copies of the plasmid [5], [6]. This increase in the copy number of the transgene results in the overproduction of the corresponding transcript and protein product, preventing an accurate analysis of the function of the gene. Episomal plasmids also unevenly segregate into daughter parasites during cell division, and thus, the parasites readily lose the plasmid unless selective pressure is maintained. These technical problems hinder the progress of the molecular analysis of *P. falciparum*; thus, a new genetic tool is required.

Through its physical association with the kinetochore, the centromere is the essential genomic region for the fidelity of chromosome segregation during nuclear division. Centromere-associated functions include sister chromatid association and separation, microtubule attachment, chromosomal movement, and the establishment of heterochromatin and mitotic check-point control [7], [8], [9]. In *P. falciparum*, the highly A/T-rich regions of each chromosome have been predicted to be centromeres [10], [11]. However, there has been no experimental evidence for their function, and thus, it has remained unclear whether these regions function as actual centromeres. Previously, we cloned the putative centromere from chromosome 5 of the rodent malaria parasite *P. berghei* based on the rodent malaria gene synteny map [12]. We showed that the plasmid harbouring the cloned centromere, called the centromere plasmid, segregated with more than 99.9% efficiency into the daughter cells during cell division, demonstrating that the cloned putative centromere functioned as an actual centromere in *P. berghei*. This result was the first functional evidence of the identity of the centromere of *Plasmodium* spp.

This efficient segregation of the centromere plasmid resulted in its stable maintenance in the parasites throughout their life cycle. In addition, the centromere plasmid was maintained as a single copy without any integration over multiple rounds of nuclear division. These unique properties, such as the efficient segregation, stable maintenance, and a low copy number, suggest that the centromere plasmid could be a useful genetic tool for the study of *P. falciparum*. In this study, we anticipated that these unique properties of the centromere plasmid could solve some current technical limitations of the genetic manipulation of *P. falciparum*, such as the low transfection efficiency, the increase of copy number of the transgene, and the uneven segregation of the introduced plasmid, and thus, we attempted to develop such a plasmid for this parasite. We first identified the common feature of the centromeres of *Plasmodium* spp., and then we constructed the centromere plasmid, pFCEN, using a centromere sequence that retained the common features. The pFCEN plasmid segregated with greater than 99% efficiency into daughter cells, showing that the incorporated centromere functioned properly as an actual centromere during cell division. We further dramatically shortened the period for the generation of transgenic parasites by using pFCEN. Based on these properties of pFCEN, we discuss how the centromere plasmid improves the current genetic modification technology of *P. falciparum*.

## Results

### Sequence analysis of the centromere of *Plasmodium* spp. and construction of the *P. falciparum* centromere plasmid

**Figure 1:**
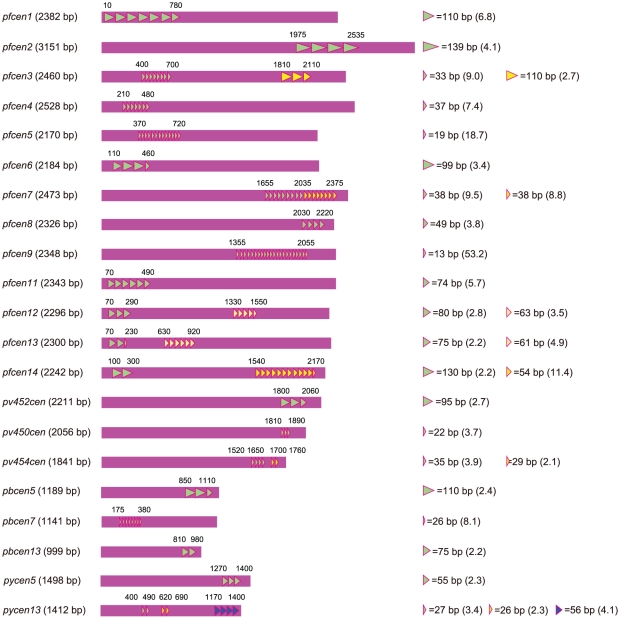
Centromere of *Plasmodium* spp. Schematic drawings of the centromeres of *Plasmodium* spp., including *P. falciparum*, *P. vivax*, *P. berghei*, and *P. yoelii* are shown. Each centromere of *P. falciparum*, *P. berghei* and *P. yoelii* is named after the corresponding chromosome number, and the centromeres of *P. vivax* are named after the corresponding contig numbers. Triangles indicate the repetitive sequence elements that were identified using the Tandem Repeats Finder program. The numbers in parenthesis show the repeat counts for each repetitive region. Other sequence information is summarised in Table S1.

The centromere of *P. falciparum* has been predicted to be a 2–3 kb AT-rich region (>96%) with no protein-coding potential in chromosomes 1 through 9, 12 and 13 [10], [11], [13]. As a result of the sequence analysis of the *P. falciparum* genome, we had previously identified two additional putative centromeres in chromosomes 11 and 14 ([12] Figure 1 and Table S1). According to these predictions, in the present study, we first amplified all of the putative centromeres. Several centromeres were cloned into the TA cloning vector (data not shown), but the centromere plasmids, including those cloned centromeres, could not be generated because of their instability in *E. coli* cells. The instability of the resulting centromere plasmids was likely caused by their high A/T contents.

To generate a stable centromere plasmid in *E. coli* cells, we next attempted to shorten the centromere. To this end, we analysed in detail the sequence features of the centromeres of *Plasmodium* spp. using the Dotlet program. Briefly, we predicted the centromeres of *P. vivax* and *P. berghei* and obtained sequence information for the centromere of *P. yoelii* from GenBank [14]. The subsequent dot matrix analysis of each centromere was performed using the centromeres from *P. falciparum*, *P. vivax*, *P. yoelii*, and *P. berghei*. As shown in Figure 1 and Table S1, except for two centromeres in *P. vivax*, all of the centromeres consisted of non-repetitive and repetitive regions. We did not identify the repetitive regions in the two abovementioned excepted centromeres, but these regions might have been missed due to low sequence complexities during the automatic sequence assembly in the sequencing of the genome. In addition, the repetitive regions appeared to localise to one end of the centromeres. Together with the high A/T contents, those two aspects were speculated to be common sequence features of the centromeres of *Plasmodium* spp.

We further compared the sequences of each centromere using the Dotlet and BLAST2 programs, but no conserved sequence motifs were found. The dot matrix analyses of the centromeres from chromosome 5 of the two rodent malaria parasites, *P. berghei* and *P. yoelii*, showed that their non-repetitive regions shared sequence similarities (Figure S1); furthermore, the lengths of the repetitive regions were roughly conserved (Figure 1 and Table S1). Similar results were obtained from the dot matrix analysis of the centromeres from chromosome 13 of the two rodent malaria parasites (Figure. S1). Interestingly, the non-repetitive regions of both centromeres of *P. berghei* were shorter than those of *P. yoelii* (Figure. S1). This result suggested that the lengths of the non-repetitive regions of the centromere were not conserved during evolution, suggesting that the truncation of this region does not affect the function of the centromere in *Plasmodium* spp.

**Figure 2:**
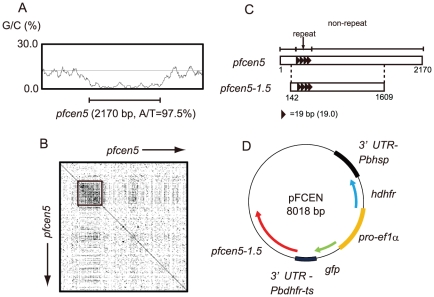
Sequence Properties of the Centromere from Chromosome 5 of *P. falciparum* and the Plasmid Map of pFCEN. (A) The sequence analysis of the centromere of chromosome 5 of *P. falciparum* was performed using Artemis 11 with regard to its length and A/T content. The line at the bottom indicates the centromere, and its length and A/T content are 2170 bp and 97.5%, respectively. The genomic sequence data of chromosome 5 were obtained from PlasmoDB (http://plasmodb.org/). (B) The repetitive region in the centromere of chromosome 5 was identified by the dot matrix analysis using the Dotlet program. The inset box indicates the repetitive region in this centromere. (C) The centromere of chromosome 5 is schematically shown and named *pfcen5* in this Figure. The line on the top indicates the repetitive and non-repetitive regions. The triangle indicates the 19 bp of the repeat sequence motif, and the number in parentheses is the number of repeats within the repetitive region. The schematic drawing of *pfcen5-1.5* also is shown. The numbers at the bottom are based on the sequence number of *pfcen5* and correspond to the beginning and the end of *pfcen5-1.5*. (D) The pFCEN plasmid is 8018 bp, and *pfcen5-1.5* is placed downstream of the 3′ UTR of the *dhfr-ts* gene of *P. berghei*.

Based on the sequence analyses of the centromeres of *Plasmodium* spp., we focused on the centromere from chromosome 5 of *P. falciparum* (Figure 2A and B) and then designed a set of primers to truncate the non-repetitive regions on both ends, as shown in Figure 2C. The amplified small centromere (1467 bp), *pfcen5-1.5*, consisted of the original repetitive region and a truncated non-repetitive region (Figure 2C); the repetitive region was placed at one end in *pfcen5-1.5*. The amplified *pfcen5-1.5* sequence was cloned into a plasmid containing the human dihydrofolate reductase (*hdhfr*) gene and the green fluorescent protein (*gfp*) gene, which served as a drug-selectable marker and a fluorescence marker, respectively (Figure 2D). The transcription of the *hdhfr* and *gfp* genes was controlled under the dual-oriented promoter of elongation factor α of *P. berghei*. The resulting centromere plasmid harbouring *pfcen5-1.5*, named pFCEN, was stably maintained in *E. coli* cells, as expected. Furthermore, the sequence analysis of the recovered plasmid showed that there were no mutations or deletions over its entire sequence (data not shown).

### *P. falciparum* can be transfected with high efficiency using pFCEN

**Figure 3:**
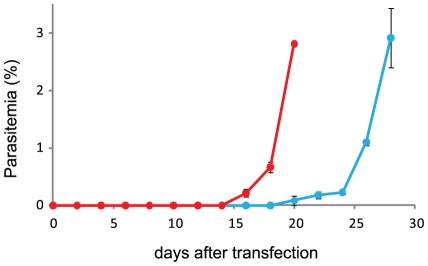
The Growth of the Transgenic Parasite after Transfection with pFCEN. The transfection of the parasite with pFCEN (red line) and pCon (blue line) were performed as described in the Materials and Methods. After 48 hours, the drug screening of the transgenic parasites was initiated by supplementing the culture medium with pyrimethamine. The parasitemia of the transgenic parasites was monitored every 48 hours after transfection. The transfection experiments for each plasmid were performed in triplicate.

The constructed pFCEN plasmid was introduced into the wild-type *P. falciparum* 3D7 strain using DNA-preloaded red blood cells (RBCs), and the parasitemia of the transfected parasites was then monitored every 48 hours. Treatment with pyrimethamine for screening the parasites that contained pFCEN was initiated 4 days after the transfection. The transfected parasite culture was diluted 2-folds with complete medium containing fresh RBCs every 10 days to compensate the loss of old RBCs. In this study, we used pCon as the negative control plasmid; this plasmid includes both the *hdhfr* and *gfp* genes but not the centromere (the *pfcen5-1.5* sequence). As shown in Figure 3, the parasites transfected with pFCEN emerged more rapidly than the control parasites carrying pCon. The parasitemia of the parasites transfected with pFCEN was approximately 2.8% at 20 days after transfection, whereas that of the control parasites was only 0.08% at the same time interval. It ultimately took 28 days for the parasitemia of the control parasites to reach 2.9%. Because the multiplication rate (every 48 hours) of the parasites carrying pCon was estimated to be 2.9 based on the parasitemia, we estimated that the transfection efficiency of pFCEN was approximately 70-fold higher than that of pCon.

The multiplication rate of the parasites carrying pFCEN was estimated to be 3.7 in this transfection experiment. Thus, the relationship between the parasitemia of each parasite at 20 days was represented by following equation:
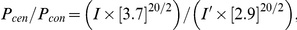
where Pcen and Pcon are the percentages of the parasitemia of parasites carrying pFCEN and pCon at 20 days after transfection, respectively, and I and I′ are the presumed initial number of the parasites transfected with those plasmids. According to this equation, the I/I′ ratio was determined to be approximately 3 using the observed Pcen and Pcon values, suggesting that pFCEN was introduced into the parasites with an efficiency similar to that of pCon. Therefore, these results suggest that the high transfection efficiency of the pFCEN plasmid was due to the improved segregation efficiency of this plasmid (discussed below), resulting in the rapid generation of transgenic parasites.

### The pFCEN plasmid segregates efficiently as the result of the function of *pfcen5-1.5* during nuclear division

During the schizogony of *P. falciparum*, a single parasite undergoes 4 to 5 rounds of DNA synthesis, mitosis, and nuclear division to produce a syncytial schizont with 16 to 22 nuclei [15]. In this process, the replicated chromosomes segregate evenly into newly generated nuclei, which result in daughter parasites, merozoites. In contrast to this even segregation, episomal plasmids that are introduced by transfection are unevenly divided into the daughter cells [16]. This uneven distribution is the reason that plasmids are rapidly lost from parasites in the absence of drug selection, leading to a growth disadvantage of the transgenic parasites in the presence of selection. As shown in Figure. S2, the parasites transfected with the pCon plasmid grew more slowly in the presence of pyrimethamine than in its absence (Method S1). In contrast, the growth of the parasites carrying the pFCEN plasmid was faster than that of the control parasite carrying pCon in the presence of this drug (Fig.S2, blue lines). This result suggests that pFCEN segregated more efficiently than pCon during nuclear division.

**Figure 4:**
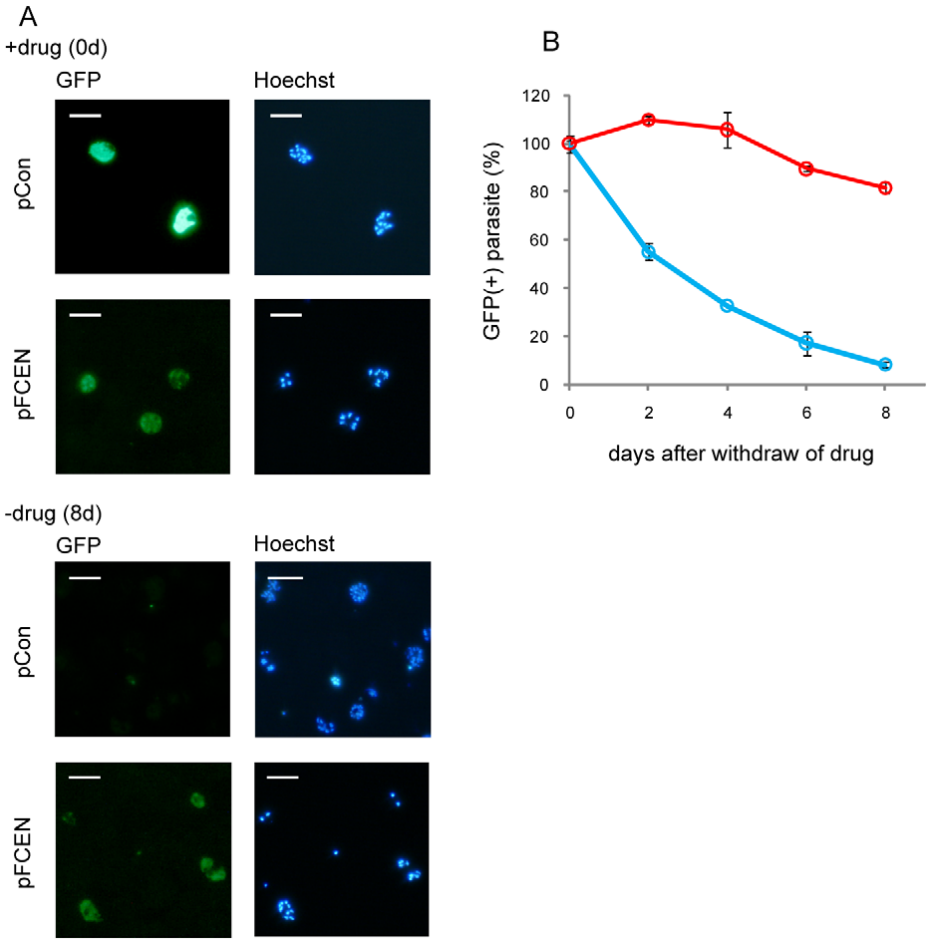
Stable Maintenance of pFCEN in the Parasites. (A) The GFP expression of the parasites transfected with pFCEN and pCon were monitored in the presence of the selective drug (upper panel) and at 8 days after the removal of the drug. The nuclei of the parasites were stained with Hoechst-33258. The scale bars indicate 10 µm. (B) The percentages of GFP-positive parasites in the absence of the drug were calculated as described in the Materials and Methods. The red and blue lines indicate the percentage of GFP-positive parasites transfected with pFCEN and pCon, respectively.

To investigate the segregation efficiency of pFCEN in detail, we next examined its stability in parasites without selective pressure using the following assay: transfected parasites were first maintained for 7 days in the presence of the drug to ensure that all of them retained the plasmid; then the drug was removed. Subsequently, the parasites were continuously cultured in the absence of the drug, and their GFP expression was monitored to determine whether the parasites retained the plasmid. This analysis showed that the GFP expression was more stably maintained in the parasites transfected with the pFCEN plasmid than in those transfected with the pCon plasmid (Figure 4A and B). Approximately 80% of the parasites carrying pFCEN still expressed GFP, whereas less than 10% of the parasites carrying pCon were GFP positive 8 days after the removal of the pyrimethamine (Figure 4B). The calculated segregation efficiencies of the pFCEN and pCon plasmids were 99.1±0.2% and 88.6±0.4% per nuclear division, respectively, based on the assumption that 5 nuclear divisions occur during schizogony. This result clearly showed that the *pfcen5-1.5* sequence conferred the improved segregation to the plasmid during schizogony, indicating that *pfcen5-1.5* functioned as a centromere in *P. falciparum*.

### The pFCEN plasmid is stably maintained as a single copy without any integration over multiple rounds of cell divisions

Interestingly, the fluorescence intensity of GFP in the parasites carrying pFCEN was significantly weaker than that in the control parasite carrying pCon (Figure 4A). This result suggested that the expression levels of GFP were different between the two types of parasites, although the transcription of the *gfp* gene was controlled by a promoter common to both plasmids. We therefore speculated that the copy numbers of these two plasmids differed, resulting in the observed difference in the GFP intensities.

**Figure 5:**
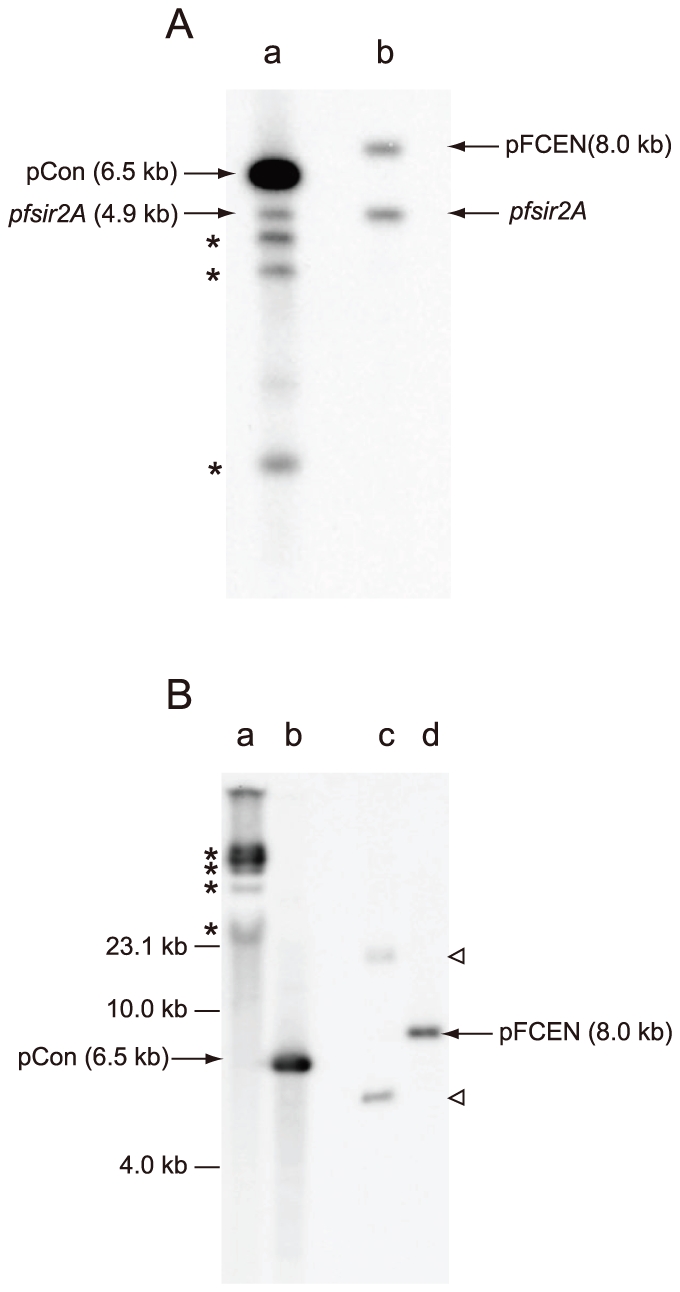
Southern Analysis of pFCEN in the Parasites. (A) To determine the copy number of pCon (lane a) and pFCEN (lane b), Southern analyses were performed by using two probe DNAs, the *gfp* and *pfsir2A* genes. The *gfp* and *pfsir2A* probes recognised the plasmids and the single, endogenous genomic *pfsir2A* copy, respectively. In this analysis, the signal from the *pfsir2A* gene was used as the internal control. The copy number of each plasmid was determined by comparing the signal intensities of the plasmids with that of the internal control. The asterisks mark three unexpected signals found in the genomic DNA including pCon. In contrast, corresponding signals were not detected in the genomic DNA including pFCEN. Therefore, these signals likely result from various derivatives, which might be irregularly generated from pCon during the replication, suggesting that *pfcen5-1.5* ensured the accurate replication of the plasmid. (B) The parasites transfected with the pFCEN and the pCon plasmids were maintained in the presence of the drug for 2 months, and their genomic DNA, including the plasmids, were purified. Southern analysis of both plasmids in the parasites was performed using purified genomic DNA and the gfp gene as a probe DNA. In this analysis, the undigested genomic DNA (lane a, pCon, and lane c, pFCEN) and the *Bam*HI-digested DNA (lane b, pCon, and lane d, pFCEN) were used. The arrows indicate the *Bam*HI-digested pFCEN and pCon. The upper and lower triangles indicate the closed circular and the super-coiled forms of pFCEN, respectively. Asterisks indicate concatemers of pCon that were detected in the undigested genomic DNA containing pCon.

To determine the copy number of the pFCEN and pCon plasmids, we conducted a Southern analysis of the genomic DNA isolated from the parasites carrying each plasmid by simultaneously using two DNA probes, one for the *gfp* gene and one for the *pfsir2A* gene. In this analysis, the *pfsir2A* gene was used as an internal standard, and the copy number of each plasmid was estimated based on the comparison of the intensities of the signals from the *gfp* and *pfsir2A* genes. As shown in Figure 5A, the signal of pCon was significantly stronger than that of pFCEN. The copy numbers of pFCEN and pCon were calculated to be 1.1±0.1 and 10.2±1.4, respectively, indicating that *pfcen5-1.5* suppressed the increase in the copy number of the plasmid.

The Southern analysis of pFCEN showed that the signal for this plasmid was detected at its expected size when the isolated genomic DNA was digested with restriction enzymes that cleaved the pFCEN once (see Materials and Methods and Figure 5A). If pFCEN was integrated into the parasite genome by single or double crossover, the signals would be detected at sizes different from that of the plasmids. Therefore, pFCEN would not be integrated into the genome of the parasites during replication, implying that it would be maintained in an episomal form in the parasite over multiple rounds of cell division.

Because pFCEN did not have any sequence homology with the genomic sequence of *P. falciparum*, except for the centromere (*pfcen5-1.5*), it would not be expected to be integrated. To exclude the possibility of integration, we next investigated whether the centromere plasmid including a DNA sequence from *P. falciparum* could be integrated during long-term cultivation. To this end, we generated another centromere plasmid, pFCENv2 (Figure. S3A, Method S2). The pFCENv2 plasmid contains the 5′-UTR of the calmodulin gene (1011 bp) and the 3′-UTRs of the heat shock protein (834 bp) and histidine-rich protein 2 (590 bp) genes of *P. falciparum* and these DNA sequences could be utilized for the single crossover recombination with the genome of *P. falciparum*. We cultured the transgenic parasites carrying pFCENv2 for 2 months in the presence of the drug and then used their genomic DNA for Southern analysis. As shown in Figure. S3B, the signals of pFCENv2 were detected at its original size when the genomic DNA was digested with a single-cleavage restriction enzyme, indicating that this plasmid was not integrated into the genome of *P. falciparum* (Method S3). We additionally performed two independent similar experiments, but did not observe any homologous recombination between pFCENv2 and the genome of the parasites (data not shown). Therefore, these results clearly showed that the pFCENv2 can be maintained as an episomal plasmid as well as the pFCEN. Because similar suppression of homologous recombination was observed with the centromere plasmid of *P. berghei* in which the recombination is more readily occurred than in *P. falciparum*, the recombination between the centromere plasmid and the genomic DNA of *P. falciparum* might be suppressed by the function of *pfcen5-1.5*.

In *P. falciparum*, plasmids replicate by a rolling circular mechanism and ultimately form large concatemeric multimers [5], [6]. To investigate whether pFCEN formed concatemers, Southern analysis was performed using the *gfp* gene as a target. Undigested genomic DNA was isolated from parasites maintained for over two months under drug selection. In this analysis, only two signals were detected at larger (18.0 kb) and smaller (5.5 kb) sizes than that of pFCEN (8.0 kb), suggesting that these two signals were from the open circular and the super-coiled forms of pFCEN, respectively (Figure 5B). In contrast to pFCEN, a strong signal was detected at more than 50 kb in the undigested genomic DNA from parasites carrying pCon (Figure 5B), indicating the formation of large concatemers, as described above. Similar Southern analysis of the control parasite showed that the size of the strong signal (concatemers) shifted to a smaller size when the genomic DNA was digested with *Bam*HI, which is a restriction enzyme that cleaves once in the pCon sequence. The detected signal size was consistent with that of pCon; thus, the high copy number was caused by the formation of large concatemers. Therefore, all of these results indicate that *pfcen*5-1.5 suppressed replication via the rolling circular mechanism.

## Discussion

In this study, we developed a centromere plasmid, pFCEN, for *P. falciparum* by incorporating a shortened centromere, *pfcen5-1.5*, into the plasmid DNA. The pFCEN plasmid segregated into daughter cells with approximately 99% efficiency during cell division and, thus, was stably maintained in the parasites without any drug-mediated selective pressure. These results clearly demonstrate that *pfcen5-1.5* functioned as a centromere in *P. falciparum*.

The sequence analysis of the centromeres of *Plasmodium* spp. suggested that they have the following common sequence features: they consist of repetitive and non-repetitive regions, and the repetitive region is located at the one end of the centromere. This analysis further suggested that the length of the repetitive regions was conserved during evolution but that the length of the non-repetitive regions was not. In addition, our previous results showed that the repetitive or non-repetitive regions alone did not fully function in the rodent malaria parasite *P. berghei* [12], indicating that both regions are required for the function of the centromere in these parasites. Taken all together, we speculate that the centromere could function properly in *P. falciparum* as long as it retained the combination of the full-length repetitive region and a portion of the non-repetitive region. Indeed, our present results showed that the small centromere *pfcen5-1.5*, which consists of the original repetitive region and a truncated non-repetitive region, functioned as a centromere in the parasites, supporting the above hypothesis. The centromeres of eukaryotes generally form centromere-specific nucleosomes, which includes the histone H3 variant, CENP-A. This centromere-specific nucleosome provides the structural basis for the assembly of the kinetochore proteins [17]. Recent reports on *Saccharomyces cerevisiae* and humans further showed that the specific chaperones, Scm3 and HUJRP, respectively, mediate the loading of CENP-A at the centromere [18], [19]. Therefore, we hypothesized that those sequence features might provide the signal for the recruitment of the centromere-specific chaperone to the centromere in *P. falciparum*. Based on those common sequence features, it will be possible to design centromeres for other *Plasmodium* spp., and similar centromere plasmids will be developed for those parasites.

The genome of *P. falciparum* has been elucidated, and the functional analysis of the genes of this organism is a major tool for understanding its biology and for the identification of drug targets and vaccine candidates. However, the function of many *P. falciparum* genes remains unclear. One of the reasons for this must be that the technology for the genetic modification of *P. falciparum* is still poor. As described in the Introduction, the gene transfer techniques for this parasite have several problems, such as a low transfection efficiency, the instability of the transgene in the parasite, and the increase in its copy number. In contrast, the pFCEN plasmid demonstrated the following technical advantages through the function of *pfcen5-1.5*. First, transgenic parasites can be quickly generated using pFCEN because of the efficient segregation in this parasite. Second, pFCEN is maintained as a single copy in the parasites as a result of its precise replication. Third, pFCEN is not integrated into the genome over multiple rounds of DNA replications and is constantly maintained as extrachromosomal DNA. These advantages will allow the improvement of the gene transfer technique for *P. falciparum*, and thus, the pFCEN5 plasmid promises to be a standard tool for the molecular genetic study of *P. falciparum*. On the other hand, the pFCEN cannot be utilized for the genome modifications of *P. falciparum*, because the homologous recombination might be suppressed by the function of the centromere. Thus the pFCEN is not suitable for the gene-knock out and the gene-knock in experiments.

*P. falciparum* develops unusual organelles, which are called the rhoptry and the microneme, and secretes the proteins that are stored in these organelles when the parasites invade host cells [20]. In addition, this parasite expresses its own proteins on the cell surface and in the cytoplasm of the host cells and utilises these proteins for the evasion of the host immune response and for the interaction with host proteins [21]. Thus, the function of these genes could be predicted based on the localisation of its product in *P. falciparum*. Currently, to investigate the localisation of the gene products in these parasites, fluorescence marker proteins, such as GFP and RFP, have been fused to the N- or C-terminus of the gene products by homologous recombination. However, this approach takes several months to generate the desired transgenic parasites because of the low recombination frequency. Furthermore, when these fusion proteins are expressed, high amounts of the recombinant proteins are expressed in the parasites because of the high copy number, resulting in their mislocalisation, as a portion of these proteins are left in the cytoplasm without being sorted. In contrast to these approaches, gene products may be easily fused to fluorescent proteins using pFCEN. As shown in this study, transgenic parasites that express fluorescence-tagged proteins may be generated within two weeks using pFCEN. In addition, the expression level of the recombinant fusion protein should be similar to that of the endogenous gene product because of the single-copy nature of the plasmid. Thus, pFCEN will be useful for the localisation analysis of proteins in *P. falciparum*.

The elucidation of transcriptional regulation mechanisms is important for understanding the biology of *P. falciparum*. Stage-specific transcription factors were recently identified in both human and rodent malaria parasites, providing clues to the transcriptional regulation mechanisms of *Plasmodium* spp. [22], [23], [24], [25], [26]. *In vivo* evaluation of the transcriptional activities of specific promoters is also necessary for studying the transcriptional regulation of the parasite. In addition, serial site-specific mutations and deletions in those regulatory sequences will allow for the identification of the cis-acting elements. However, the promoters on conventional plasmids (e.g., pCon) possess an apparent high activity because of their high copy number, and thus, it would be difficult to use these plasmids for such gene expression profiling. Moreover, a significant amount of time and effort would be required to generate transgenic parasites carrying serial site-specific mutations and deletions by homologous recombination. Because pFCEN is maintained as a single-copy plasmid in the parasites, transcription from its promoter should be similar to the endogenous expression with regard to the timing and activity level. In addition, transgenic parasites can be generated more quickly using pFCEN than using homologous recombination, allowing the high-throughput analysis of cis-acting elements. Therefore, we propose that pFCEN will be the best tool for profiling gene expression in *P. falciparum*.

We showed that the shortened form of the centromere functioned properly in *P. falciparum* and that the centromere plasmid pFCEN can be utilised as a new genetic tool for the study of this parasite. Because the centromeres of *Plasmodium* spp. exhibit a common sequence structure, similar centromere plasmids can be constructed for other species, such as *P. vivax*, for which genetic modification technologies have been under development. In addition, a linear form of the artificial chromosome for *P. falciparum* can be developed by incorporating the telomere sequence into pFCEN. Such an artificial chromosome will be a useful tool for epigenetic studies and chromosome biology research focused on this parasite.

## Materials and Methods

### Sequence analysis of the Centromere of *Plasmodium* spp

The sequences of the predicted centromeres of chromosomes 1 through 9, 12 and 13 of *P. falciparum* were obtained from the public database PlasmoDB (http://plasmodb.org). The centromeres of chromosome 11 and 14 of *P. falciparum* were predicted based on their A/T contents. Briefly, the entire genomic sequences of both chromosomes were scanned with regard to the A/T content using Artemis 11 with a window size of 120 bp (http://www.sanger.ac.uk/resources/software/artemis/) and highly A/T-rich regions (>95.0%) were identified as putative centromeres. The centromeres of *P. vivax* and *P. berghei* were predicted based on the genomic synteny conserved between *Plasmodium* spp. First, the orthologues of genes up-stream and down-stream of the centromere of *P. falciparum* were identified by searching by sequence similarity, and the sequences of the genomic regions between the up-stream and down-stream orthologous genes were then obtained. Next, the highly A/T-rich regions in the obtained genomic sequences were identified in a similar manner as described above and then predicted to be the putative centromeres of *P. vivax* and *P. berghei*. The sequences of the centromeres from chromosome 5 (DQ054838.1) and 13 (DQ054839.1) of *P. yoelii* were obtained from GenBank (www.ncbi.nlm.nih.gov/sites/entrez?db=nucleotide). To identify the repetitive regions within all of the centromeres, dot matrix analysis was conducted using Dotlet (http://myhits.isb-sib.ch/cgi-bin/dotlet) with a window size of 15 bp. In these analyses, the sequence identities were only recorded when they were greater than 80% in the window. The presence and locations of sequence elements in each repetitive region were identified using the Tandem Repeats Finder program (http://tandem.bu.edu/trf/trf.html). The sequences and arrangements of any repetitive regions within the centromere were characterised using the default settings.

### Construction of the Centromere Plasmid

The DNA fragment containing the shortened centromere of chromosome 5 of the 3D7 strain of *P. falciparum* was amplified by two steps of PCR. The primary PCR was performed using the primer pair P1: 5′-GCAATAACATAATAATAGATCAATATAAC-3′ and P2: 5′-ATTAAATAATAATAACAAACGGGATAAG-3′. After the purification of the primary PCR product, the secondary PCR was performed using the purified product as the template DNA and the primer pair P3: 5′-AAAGGATCCTATAATAATTTATGTATAATTAAATTAAATATTATAAAC ACAC-3′ and P4: 5′-AAAGCGGCCGCAATTAAATATTATTTAATTATTAATATATTAATTATTTAGAC-3′. The obtained final PCR product was digested with *Bam*HI and *Not*I and then cloned into the plasmid vector containing the human dihydrofolate reductase (*hdhfr*) gene and the *gfp* gene, the products of which served as a drug-selectable marker and a fluorescence marker, respectively. The transcription of both genes was controlled under the dual-driven *eef1*-α promoter of *P. berghei*. The resulting plasmid was named pFCEN and was used as the centromere plasmid.

### Cultivation and Synchronisation of the Parasite

The *Plasmodium falciparum* 3D7 strain was obtained from the Malaria Research and Reference Reagent Resource Center (http://www.mr4.org/). The obtained parasites were cultivated with human red blood cells (RBCs: type O blood, Ht 2%, the Japanese red cross aichi blood centre) in complete medium, which consisted of RPMI-1640, containing 10% human serum (the Japanese red cross aichi blood centre), 10% AlbuMAX I (GIBCO BRL), 25 mM HEPES, 0.225% sodium bicarbonate, and 0.38 mM hypoxanthine supplemented with 10 µg/ml gentamicin, and were incubated under low-oxygen conditions (90% N_2_, 5% CO_2_, and 5% O_2_). When the parasitemia had reached approximately 5%, the infected RBCs were transferred to freshly prepared RBCs. The parasites were synchronised using 5% D-sorbitol according to a standard protocol [27]. Briefly, infected RBCs were quickly suspended in 10 volumes of 5% D-sorbitol and then incubated exactly at 37°C for 8 min. After the incubation, the parasites were collected by centrifugation, washed with incomplete medium (RPMI-1640 containing 25 mM HEPES, 0.225% sodium bicarbonate, and 0.38 mM hypoxanthine supplemented with 10 µg/ml gentamicin) and cultivated in complete medium, as described above.

### Transfection of Parasites with the Centromere plasmid Using DNA-Loaded RBCs

The transfection of the parasites was performed by the invasion of DNA-loaded erythrocytes [28]. Briefly, RBCs were washed three times with incomplete medium and resuspended to 50% hematocrit. The pFCEN and the pCon plasmids were purified using the QIAGEN miniprep kit (QIAGEN), precipitated with ethanol, and dissolved in incomplete cytomix (120 mM KCl, 0.15 mM CaCl_2_, 2 mM EGTA, 5 mM MgCl_2_, 10 mM K_2_HPO_4_/KH_2_PO_4_, 25 mM HEPES, pH 7.6). The washed RBCs and 12.5 µg of the DNA samples were combined in an ice-cold cuvette and then electroporated using a Bio-Rad Gene Pulser II at 0.31 kV and 950 µF. The DNA-loaded RBCs were immediately washed three times with complete medium and then infected with the parasites. The medium was changed every 24 hours, and the drug selection of the transgenic parasites using pyrimethamine (2.5 ng/ml) was initiated 4 days after the electroporation.

### Assay to Determine the Maintenance of the Centromere Plasmid in the Parasites

Transfected parasites were preliminary maintained for a period of 7 days in complete medium supplemented with pyrimethamine (2.5 ng/ml) to ensure that all of the parasites retained the introduced plasmids. After this period, the parasites were synchronised using 5% D-sorbitol, as described above, and then cultured in complete medium with fresh RBCs in the absence and the presence of pyrimethamine. Because the transcriptional activity of the *eef1*-α promoter is maximal in the parasites at the schizont stage, the GFP expression in was monitored by fluorescence microscopy at this developmental stage, that is, every 48 hours, after staining with Hoechst-33258 (10 µM final concentration). Similar monitoring was conducted using flow cytometry (LSR Fortessa cell analyser, BD Science). Some of the parasites died spontaneously during the *in vitro* culture, and to account for its effect, the percentage of the GFP-positive parasites in the absence of selection was expressed as a relative value, setting the percentage in the presence of the drug to 100%. Triplicate experiments were performed for each transfected parasite. Based on the percentage of the GFP-positive parasites after the removal of pyrimethamine, the segregation efficiency of the plasmid per nuclear division was calculated according to the following equation:
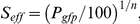
where Seff and Pgfp are the segregation efficiency and the percentage of GFP-positive parasites, respectively, and n is the number of nuclear divisions. In this study, we assumed that 5 rounds of nuclear division occurred during one blood-stage schizogony of 48 hours. We determined the segregation efficiencies of pFCEN and pCon at days 6 and 8 after the removal of drug selection and then calculated their averages.

### Southern Analysis of the Centromere Plasmid in the Parasites

The parasites transfected with pFCEN or pCon were maintained in the presence of pyrimethamine for 1 month and used for the extraction of genomic DNA. The parasite genomic DNA containing the plasmids was digested with *Nhe*I, which cleaved a single site, and blotted onto a membrane. In this Southern hybridisation, we simultaneously used both the *gfp* and *pf-sir2A* genes (PF13_0152) as probe DNAs. The *pf-sir2A* genes specifically hybridised with the parasite genome and, thus, were used as an internal control to determine the copy number of the plasmid. The signal intensities were quantified using Quantity One software (BioRad). The copy number of the pFCEN plasmid was estimated by comparing the intensity of the signal from the pFCEN plasmid with that from the parasite genome.

To determine whether the plasmids formed concatemers in the parasites over long culture periods, the parasites were maintained under drug selection for 2 months, and their genomic DNA, including the plasmids, was used for the Southern analysis. Undigested genomic DNA and *Bam*HI-digested genomic DNA were transferred to the membrane, and the plasmids were detected by hybridising with the *gfp* gene used in the above analysis.

## Supporting Information

Method S1 Growth of transgenic parasites in the presence and absence of the selective drug.
(DOC)

Method S2 Construction of pFCENv2 and the generation of the transgenic parasites carrying pFCEN5v2.
(DOC)

Method S3 Southern analysis of pFCENv2 in the parasites.
(DOC)

Figure S1 Dot matrix analyses of the centromere of two rodent malaria parasites, *P. berghei* and *P. yoelii*, were performed using the Dotlet program. In these analyses, the centromeres of chromosomes 5 and 13 of each parasite were used. The panels depict the graphical results of the matrix analysis of one centromere aligned against the other centromere in the corresponding chromosomes. The diagonal line within each analysis represents the sequence identity between the two centromeres from *P. berghei* and *P. yoelii*. The triangles indicate the repetitive region in each centromere. The truncated regions in the non-repetitive region of the centromeres from chromosomes 5 and 13 of *P. yoelii* were predicted, as shown by the white boxes.
(EPS)

Figure S2 The growth of the parasites carrying pFCEN and pCon were monitored in the presence and absence of pyrimethamine. The open and closed circles indicate the parasitemia of the parasites carrying pFCEN and pCon, respectively. The blue and red lines show the conditions in the presence and the absence of the drug, respectively.
(EPS)

Figure S3 (A) The pFCENv2 plasmid is 8477 bp, and *pfcen5-1.5* is placed downstream of the 3′ UTR of the *hrp2* gene of *P. falciparum*. In this plasmid, blasticidin S deaminase is used as the drug-selectable marker. (B) The parasites carrying pFCENv2 (lanes a and b) were maintained in the presence of the drug for 2 months, and genomic DNA was purified. Southern analysis of pFCENv2 was performed using the *gfp* gene as a probe. The arrows indicate the signals of pFCENv2. Their sizes are consistent with that of pFCENv2, indicating that the plasmid did not integrate into the parasite genome during long-term culture. (C) To determine the copy numbers of pConv2 (lane a) and pFCENv2 (lane b and c) in the parasites, Southern analyses were performed using the 3′UTR of the *hrp*2 gene of *P. falciparum* as a probe. The copy number of each plasmid was determined by comparing the signal intensities of the plasmids with that of the internal control. The copy numbers of pFCENv2 and pConv2 were estimated to be 1.1 and 3.9, respectively. The arrows indicate the signals from each plasmid and the genomic DNA of the parasites.
(EPS)

Table S1 Sequence properties of centromeres of *Plasmodium* spp including *P. falciparum*, *P. vivax*, *P. berghei* and *P. yoelii* are summarized.
(XLSX)

## References

[1] Enserink M (2010). Malaria's drug miracle in danger.. Science.

[2] Wu Y, Sifri CD, Lei HH, Su XZ, Wellems TE (1995). Transfection of Plasmodium falciparum within human red blood cells.. Proc Natl Acad Sci U S A.

[3] Waterkeyn JG, Crabb BS, Cowman AF (1999). Transfection of the human malaria parasite Plasmodium falciparum.. Int J Parasitol.

[4] Wu Y, Kirkman LA, Wellems TE (1996). Transformation of Plasmodium falciparum malaria parasites by homologous integration of plasmids that confer resistance to pyrimethamine.. Proc Natl Acad Sci U S A.

[5] O'Donnell RA, Preiser PR, Williamson DH, Moore PW, Cowman AF (2001). An alteration in concatameric structure is associated with efficient segregation of plasmids in transfected Plasmodium falciparum parasites.. Nucleic Acids Res.

[6] Williamson DH, Janse CJ, Moore PW, Waters AP, Preiser PR (2002). Topology and replication of a nuclear episomal plasmid in the rodent malaria Plasmodium berghei.. Nucleic Acids Res.

[7] Morris CA, Moazed D (2007). Centromere assembly and propagation.. Cell.

[8] Cleveland DW, Mao Y, Sullivan KF (2003). Centromeres and kinetochores: from epigenetics to mitotic checkpoint signaling.. Cell.

[9] Pluta AF, Mackay AM, Ainsztein AM, Goldberg IG, Earnshaw WC (1995). The centromere: hub of chromosomal activities.. Science.

[10] Bowman S, Lawson D, Basham D, Brown D, Chillingworth T (1999). The complete nucleotide sequence of chromosome 3 of Plasmodium falciparum.. Nature.

[11] Hall N, Pain A, Berriman M, Churcher C, Harris B (2002). Sequence of Plasmodium falciparum chromosomes 1, 3–9 and 13.. Nature.

[12] Iwanaga S, Khan SM, Kaneko I, Christodoulou Z, Newbold C (2010). Functional identification of the Plasmodium centromere and generation of a Plasmodium artificial chromosome.. Cell Host Microbe.

[13] Gardner MJ, Tettelin H, Carucci DJ, Cummings LM, Aravind L (1998). Chromosome 2 sequence of the human malaria parasite Plasmodium falciparum.. Science.

[14] Kooij TW, Carlton JM, Bidwell SL, Hall N, Ramesar J (2005). A Plasmodium whole-genome synteny map: indels and synteny breakpoints as foci for species-specific genes.. PLoS Pathog.

[15] Gerald N, Mahajan B, Kumar S (2011). Mitosis in the human malaria parasite Plasmodium falciparum.. Eukaryot Cell.

[16] van Dijk MR, Vinkenoog R, Ramesar J, Vervenne RA, Waters AP (1997). Replication, expression and segregation of plasmid-borne DNA in genetically transformed malaria parasites.. Mol Biochem Parasitol.

[17] Perpelescu M, Fukagawa T (2011). The ABCs of CENPs.. Chromosoma.

[18] Xiao H, Mizuguchi G, Wisniewski J, Huang Y, Wei D (2011). Nonhistone Scm3 binds to AT-rich DNA to organize atypical centromeric nucleosome of budding yeast.. Mol Cell.

[19] Barnhart MC, Kuich PH, Stellfox ME, Ward JA, Bassett EA (2011). HJURP is a CENP-A chromatin assembly factor sufficient to form a functional de novo kinetochore.. J Cell Biol.

[20] Cowman AF, Crabb BS (2006). Invasion of red blood cells by malaria parasites.. Cell.

[21] Goldberg DE, Cowman AF (2010). Moving in and renovating: exporting proteins from Plasmodium into host erythrocytes.. Nat Rev Microbiol.

[22] De Silva EK, Gehrke AR, Olszewski K, Leon I, Chahal JS (2008). Specific DNA-binding by apicomplexan AP2 transcription factors.. Proc Natl Acad Sci U S A.

[23] Yuda M, Iwanaga S, Shigenobu S, Mair GR, Janse CJ (2009). Identification of a transcription factor in the mosquito-invasive stage of malaria parasites.. Mol Microbiol.

[24] Yuda M, Iwanaga S, Shigenobu S, Kato T, Kaneko I (2010). Transcription factor AP2-Sp and its target genes in malarial sporozoites.. Mol Microbiol.

[25] Flueck C, Bartfai R, Niederwieser I, Witmer K, Alako BT (2010). A major role for the Plasmodium falciparum ApiAP2 protein PfSIP2 in chromosome end biology.. PLoS Pathog.

[26] Campbell TL, De Silva EK, Olszewski KL, Elemento O, Llinas M (2010). Identification and genome-wide prediction of DNA binding specificities for the ApiAP2 family of regulators from the malaria parasite.. PLoS Pathog.

[27] Lambros C, Vanderberg JP (1979). Synchronization of Plasmodium falciparum erythrocytic stages in culture.. J Parasitol.

[28] Deitsch K, Driskill C, Wellems T (2001). Transformation of malaria parasites by the spontaneous uptake and expression of DNA from human erythrocytes.. Nucleic Acids Res.

