# Supplementary material for: Centromere Plasmid: A New Genetic Tool for the Study of Plasmodium falciparum
Source: PLoS One. 2012 Mar 30;7(3):e33326. doi: 10.1371/journal.pone.0033326 (PMC3316556; doi:10.1371/journal.pone.0033326)
Supplement: Method S1 — Growth of transgenic parasites in the presence and absence of the selective drug. (DOC) [file pone.0033326.s001.doc]

**Method S1. Growth of transgenic parasites in the presence and absence of the selective drug.**

The growth of the parasites carrying pFCEN and pCon was examined in the presence and the absence of pyrimethamine. The parasitemia of those parasites were determined every 24 hours. In this assay, the initial parasitemia of the parasites carrying pFCEN and pCon were 0.05 % at day 0.
