# Supplementary material for: Centromere Plasmid: A New Genetic Tool for the Study of Plasmodium falciparum
Source: PLoS One. 2012 Mar 30;7(3):e33326. doi: 10.1371/journal.pone.0033326 (PMC3316556; doi:10.1371/journal.pone.0033326)
Supplement: Method S2 — Construction of pFCENv2 and the generation of the transgenic parasites carrying pFCEN5v2. (DOC) [file pone.0033326.s002.doc]

**Method S2. Construction of pFCENv2 and the generation of the transgenic parasites carrying pFCEN5v2.** The pfLN-ENR-GFP sequence was obtained from the Malaria Research and Reference Reagent Resource Center (http://www.mr4.org/) and used for the construction of pFCENv2. The gene encoding the GFP-fused enoyl-acyl carrier protein reductase was replaced with the *gfp* gene in the pfLN-ENR-GFP plasmid, and then *pfcen5-1.5* was cloned into the *Eco*RI site of this modified plasmid. The resulting centromere plasmid, pFCEN5v2 (8477 bp), was introduced into *P. falciparum* in a similar manner as that described in the Materials and Methods. Drug screening of the transgenic parasites using blasticidin S (2.5 μg/ml) was initiated 4 days after the electroporation.
