# Supplementary material for: Centromere Plasmid: A New Genetic Tool for the Study of Plasmodium falciparum
Source: PLoS One. 2012 Mar 30;7(3):e33326. doi: 10.1371/journal.pone.0033326 (PMC3316556; doi:10.1371/journal.pone.0033326)
Supplement: Method S3 — Southern analysis of pFCENv2 in the parasites. (DOC) [file pone.0033326.s003.doc]

**Method S3. Southern analysis of pFCENv2 in the parasites.** Southern analysis of pFCENv2 was performed essentially as described for pFCEN in the Materials and Methods section. To estimate the copy number of pFCENv2, the genomic DNA isolated from transgenic parasites carrying pFCENv2 was digested with *Eco*RV and *Sca*I and hybridised with the DNA fragment of the 3’ -UTR of the histidine-rich protein 2 (ID#: MAL7P.1.231) of *P. falciparum*. This probe can simultaneously detect pFCENv2 and the genomic region; thus, the copy number of pFCENv2 was estimated by comparing their signal intensities. To investigate whether pFCENv2 was stably maintained as an episomal plasmid in the parasites, the genomic DNA was isolated from the parasites, digested with *Sca*I, and hybridised with the *gfp* gene.
